# *BKM-react*, an integrated biochemical reaction database

**DOI:** 10.1186/1471-2091-12-42

**Published:** 2011-08-08

**Authors:** Maren Lang, Michael Stelzer, Dietmar Schomburg

**Affiliations:** 1 Department of Bioinformatics and Biochemistry, Institute for Biochemistry and Biotechnology, Technische Universität Braunschweig, Langer Kamp 19 B, 38106 Braunschweig, Germany

## Abstract

**Background:**

The systematic, complete and correct reconstruction of genome-scale metabolic networks or metabolic pathways is one of the most challenging tasks in systems biology research. An essential requirement is the access to the complete biochemical knowledge - especially on the biochemical reactions. This knowledge is extracted from the scientific literature and collected in biological databases. Since the available databases differ in the number of biochemical reactions and the annotation of the reactions, an integrated knowledge resource would be of great value.

**Results:**

We developed a comprehensive non-redundant reaction database containing known enzyme-catalyzed and spontaneous reactions. Currently, it comprises 18,172 unique biochemical reactions. As source databases the biochemical databases *BRENDA*, *KEGG*, and *MetaCyc *were used. Reactions of these databases were matched and integrated by aligning substrates and products. For the latter a two-step comparison using their structures (*via InChIs*) and names was performed. Each biochemical reaction given as a reaction equation occurring in at least one of the databases was included.

**Conclusions:**

An integrated non-redundant reaction database has been developed and is made available to users. The database can significantly facilitate and accelerate the construction of accurate biochemical models.

## Background

For the construction of cellular models, the development of organism-specific reaction networks is essential. A number of sources for biochemical reactions exist, as the databases *BRENDA *[1], *KEGG *[2], and *MetaCyc *[3]. In general, the integration of biological databases is not trivial [4]. Due to the fact that the completeness of reaction data differs between the databases, it becomes important to combine the available reaction information of the used source databases in form of an integrated reaction database.

So a combination will lead to more complete and reliable metabolic networks. Therefore it is necessary to find identical reactions between the recognized databases. As different compound names and compound IDs, as well as reaction IDs, are in use within the described biochemical reactions a comparison is far from straightforward. A major obstacle results from the use of generic compound names, *e.g. 'an aldehyde' *or *'an alcohol'*. Furthermore some reactions even occur in the same database twice with different reaction IDs.

Integrated databases exist for diverse biological topics. The *TRANSPATH^® ^*database for example is an integrated database which deals with signal transduction information [5]. As an example for an integrated metabolic database system the database *BioSilico *can be mentioned here [6]. For creation of this database, information of the metabolic databases *KEGG*, *ENZYME *[7], *EcoCyc *[8], and *MetaCyc *was combined, the latter two building parts of *BioCyc *[9]. The database *BioSilico *includes information on enzymes, biochemical compounds, and reactions. Radrich *et al. *[10] provide a semi-automated tool for the process of genome-scale network reconstruction demonstrated on the basis of data for *Arabidopsis thaliana*. Their integrated data set is built on the two sources *KEGG *and *AraCyc *[11]. Furthermore a reaction database on human biological pathways and processes named *Reactome *[12] exists as well as an annotated reaction database called *Rhea *[13], basically a modified version of the reactions defined in the *IUBMB *enzyme list [14]. A collection of biochemical reactions and pathways in printed form contains the book *Biochemical Pathways: An Atlas of Biochemistry and Molecular Biology *[15].

## Methods

In this work information from the biological databases *BRENDA *[1], *KEGG *[2], and *MetaCyc *[3] was used (May 2011). Reaction comparisons were done by an *in silico *approach in which two steps, first a comparison of reactant structures using *InChIs *(linearized chemical structure descriptors [16]) and, second, a compound name comparison (incl. synonyms), were combined. An *InChI *structure coding was generated based on an original *Molfile *(contains molecular structure information [17]) by using a special converting tool (*InChI version 1 (software version 1.03) for Standard and Non-Standard InChI/InChIKey *[18]). By using only relevant layers of an self-generated *InChI*, a higher matching rate was achieved. For this purpose we dropped the *InChI *layers dependent on the ionisation state so that *e.g. *acetic acid and the acetate ion were considered to be the same compound. Reactions without *EC *numbers were included as well as those reactions with incomplete *EC *numbers. Spontaneous reactions without *EC *number were labelled *SPONTANEOUS*. Before the comparison, the compounds water (H_2_O) and proton (H^+^) were removed from the reactions. Additionally, a stoichiometry check was executed. This information was added as attribute to the reactions in the database as a quality measure. Stoichiometrically imbalanced reactions were marked as *incomplete *in the column *Stoichiometry*, except in cases where only a proton or water is missing. In two supplemental columns the incomplete cases are differentiated into *Missing Substrate *and *Missing Product*.

For the compound name based comparison step all found synonyms were used as well as generated '*DAYLIGHT names*' (*Chemical Information Systems, Inc*. [19]). We applied a special conversion that removed most of the common sources of differences in equivalent compound names like hyphens, parentheses, *etc*. Most of the special characters, except '+' and apostrophe ('), were deleted. For identifying common reactions, all available synonyms and '*DAYLIGHT names*' (see above) of the compounds are included in form of a link table containing assigned compound IDs. Where possible, *KEGG *glycan IDs (*G *number) were exchanged by their corresponding compound IDs (*C *number). Reactions with NAD(P)/H (*BRENDA*) and NADP/H_OR_NO_P (*MetaCyc*) were split into two reactions, one with NADH, the other with NADPH. The reaction ID of the form without phosphate was labelled as the original but with *_WOP *(= WithOut Phosphate) at the end.

Data download, storage, and comparison was realized by *C++ *as well as *Python *scripts and embedded *MySQL *statements. By executing a cron-job in regular time points, the information about metabolites, enzymes, reactions, *Molfiles*, and *InChIs *was downloaded from the source databases and so kept up to date automatically.

**Figure 1:**
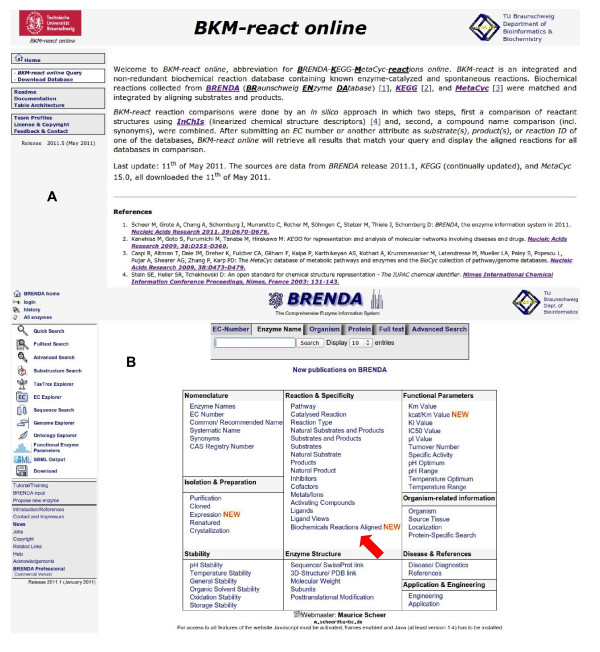
**Link to the integrated non-redundant reaction database**. Website of *BKM-react *(A) and the *BRENDA *main menu with the link *Biochemicals Reactions Aligned*(B, see arrow).

**Figure 2:**
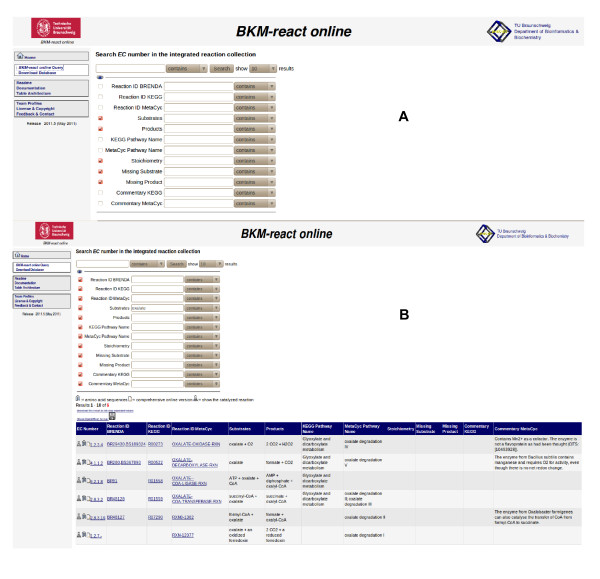
***BKM-react***. Complete menu for the *MySQL *query of the reaction table (A) and example query (B).

The access to the integrated database is possible *via *the link to *BKM-react *[20], Figure 1A, or *via *the *BRENDA *website, making use of the *BRENDA *query engine. Figure 1B illustrates the access to the integrated non-redundant reaction database [21] → *Reaction & Specificity → Biochemicals Reactions Aligned *(see arrow). Parameters for doing queries are presented in Figure 2A for the reaction table. Figure 2B shows an example for a query result. The downloadable content of the database consists of three tables, containing the compared reactions, the according compounds as well as a link table connecting both with each other.

## Results and discussion

The combined database contains a unique list of reactions that occur in any of the compared databases *BRENDA *[1], *KEGG *[2], and *MetaCyc *[3] and the associations between equivalent reactions. Additionally these reactions are assigned to *KEGG *and *MetaCyc *pathways. Table 1 lists the data used for the comparison. The largest number of reactions originates from the *BRENDA *database, followed by *MetaCyc*, and *KEGG*.

**Table 1:** Overview reaction sources and data

	Different *EC* numbers	Incomplete *EC* numbers	Reaction IDs	Reaction IDs without *EC* number	Compound IDs	Synonyms	*Molfiles*	*InChIs*
***KEGG***	3,761	122	8,452	1,288	6,522	11,597	6,327	5,416
***MetaCyc***	4,159	138	9,343	2,236	6,095	19,707	6,035	5,782
***BRENDA***	4,425	207	10,109	55	9,750	20,922	9,750	5,242

The number of reactions in *BRENDA *is in fact close to 180,000. In this case only complete reactions with natural substrates were included.

A significantly improved matching of reactions was achieved by removing the compounds H^+ ^and water (H_2_O) from the reactions before comparing them because the reactions in the databases are not always stoichiometrically balanced. The order of executing first the *InChI *comparison followed by the name comparison was chosen because identical synonyms may occur for different compounds. To rely on synonyms could therefore result in incorrect links. By using the reverse order more false positive matchings would appear.

**Figure 3:**
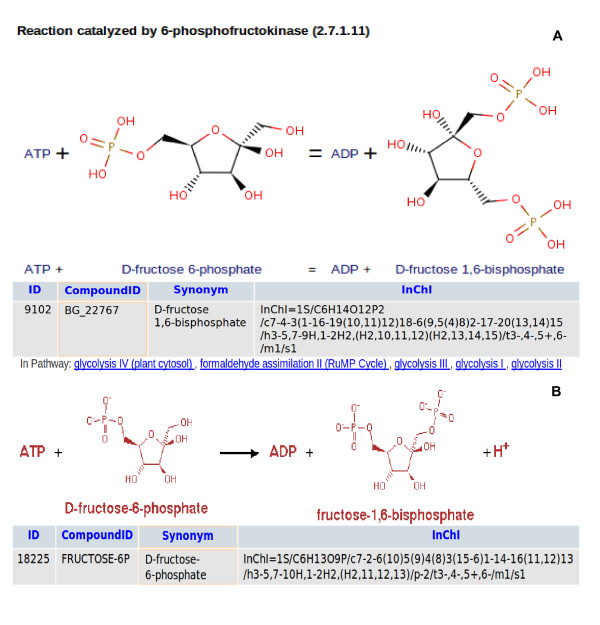
**Screenshots of the databases *BRENDA *and *MetaCyc***. These reactions, *BRENDA *reaction *BR47724 *and the *MetaCyc *reaction *6PFRUCTPH*O*S-RXN *are matching the *KEGG *reaction *R04*7*79 *(Fig. 4 B) because of the complete *InChI *string for *beta-D*-Fructose 1,6-bisphosphate even if *MetaCyc *names it *D*-fructose-6-phosphate.

**Figure 4:**
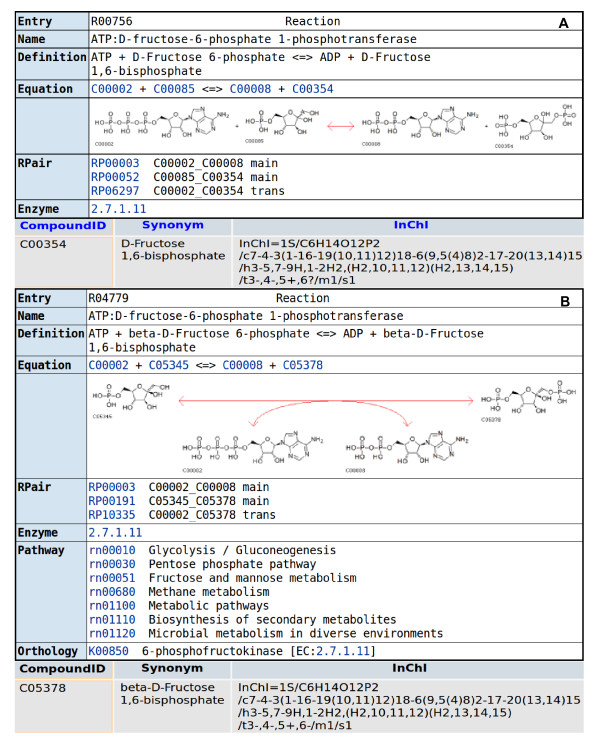
**Screenshots of the database *KEGG***. *KEGG R00756 *and *R04779*. The second reaction is the preferred one. *C04779 *possesses the complete *InChI *string and is therefore matched with the more complete described metabolites of the other databases.

One of the difficulties in the comparison consists in the - sometimes implied - stereochemistry not given in the compound name. Whereas cases like "alanine" being used for "*L*-alanine" are obviously to be expected, sometimes things become more complicated. For example, in *BRENDA *and *MetaCyc beta*-stereochemistry is implied for C5 of *D*-fructose-1,6-bisphosphate, being the major stereoisomer (see Figure 3A and 3B), the *KEGG *database includes in fact two different reactions, one with *beta*-stereochemistry at C5, the other with undefined stereochemistry (see Figure 4A and 4B) where pathway information is only assigned to the reaction with the full stereochemistry. In general metabolites with complete stereochemistry are favored in *BKM-react. *

If no structural information is available, reactions are allowed to match by name comparison.

This example shows a general problem in biochemical compound name comparison. The large majority of biochemists refer to *S*-alanine just by the name alanine although the name in principle is ambiguous or should be used for the racemate. In most cases we assume that for the standard amino acids the name without stereo-descriptor implicitly means *S*- (or *L*-, respectively). This holds true also for some other compound names where the stereo-descriptor is implicitly given. A related problem occurs at positions where the stereochemistry is ambiguous like in the case of C1 of *D*-glucose. In some cases the stereochemistry for this position is undefined in the *Molfiles *[17], in others the more stable form (*e.g. beta *in the case of glucose) is used and defined.

Although all three databases offer their own *InChIs*, they are not directly comparable because *KEGG *uses the non-standard form of an *InChI*, whereby *MetaCyc *and *BRENDA *use the standard *InChI *format. So for a standardized comparison it is necessary to use self-generated *InChIs *based on *Molfiles*. For this purpose the official *IUPAC *converting tool was utilized [18]. A higher matching rate was achieved by using only essential layers (see *Methods *section) of an *InChI *string. A drawback is that not for each compound an *InChI *is available, *e.g. *for macromolecular reactants or for generic compounds.

**Figure 5:**
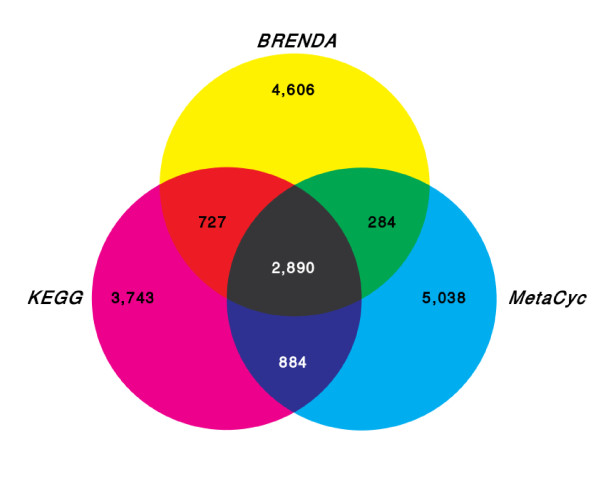
**Distribution of unique reactions between the used databases**.

A pairwise comparison of reactions revealed a high identity between *KEGG *&*MetaCyc*. About 50% reactions were equal, out of which most were also found in *BRENDA *(Figure 5). Between *MetaCyc *&*BRENDA *3,174 reactions were identified to be equal. Comparing *KEGG *&*BRENDA*, even more reactions (3,617) could be assigned to each other.

Table 2 shows the assignment of diverse reactions between the databases which are equal. There are examples of reactions that have a 1:n relation because of redundant reactions occurring within the same database. In *KEGG *for example, metabolites are differentiated into glycans and compounds, respectively. This means that identical compounds may get two different IDs, starting with *G *and *C*. This results in reactions with different reaction IDs (no. 3 in Tab. 2). Sometimes there are synonyms or keto-enol tautomers which describe one reaction in various forms (no. 1 in Tab. 2) or other alternative writing styles (no. 2 in Tab. 2). Further *KEGG *uses one reaction-ID for the same reaction being catalysed by enzymes with different *EC *numbers, whereas *MetaCyc *often uses different reaction IDs in such cases (no. 1 in Tab. 2).

**Table 2:** Some instructive cases of different forms for identical reactions

No.	*KEGG*	*MetaCyc*	*BRENDA*	Definition
1	*R04915*			Quinoline-3,4-diol + Oxygen <=> Formylanthranilate + CO
	*R05719*			3-Hydroxy-1H-quinolin-4-one + Oxygen <=> Formylanthranilate + CO
		*1.13.11.47-RXN*		3-hydroxy-1H-quinolin-4-one + oxygen = carbon monoxide + *N*-formylanthranilate
			*BR22597*	3-hydroxy-1H-quinolin-4-one + O2 = N- formylanthranilate + CO

2	*R00004*			Diphosphate + H2O <=> 2 Orthophosphate
		*INORGPYROPHOSPHAT-RXN*		diphosphate + H_2_O = 2 phosphate + H^+^
			*BR22749*	diphosphate + H2O = 2 phosphate

3	*R00010*			alpha, alpha-Trehalose + H2O <=> 2 D-Glucose (*C01083*)
	*R06103*			Trehalose + H2O <=> 2 D-Glucose (*G00293*)
		*TREHALA-RXN*		trehalose + H2O → 2 β-D-glucose
			*BR15991*	alpha, alpha-trehalose + H2O = 2 D-glucose
			*BS370856*	alpha, alpha-trehalose + H2O = beta-Dglucose

In Figure 5 the distribution of equal reactions occurring in any of the three databases is illustrated. 2,890 of all reactions are contained in all three databases, corresponding to 34% of all *KEGG *reactions, 31% of all *MetaCyc *reactions, and 29% of the included *BRENDA *reactions, respectively. In the present version of the data set, 3,743 *KEGG *reactions, 5,038 *MetaCyc *reactions, and 4,606 *BRENDA *reactions occur only in the respective database (Figure 5). Altogether the non-redundant reaction database up to now contains 18,172 unique reactions and 20,358 *EC*/reaction combinations as some reactions are catalyzed by a number of different enzymes.

**Figure 6:**
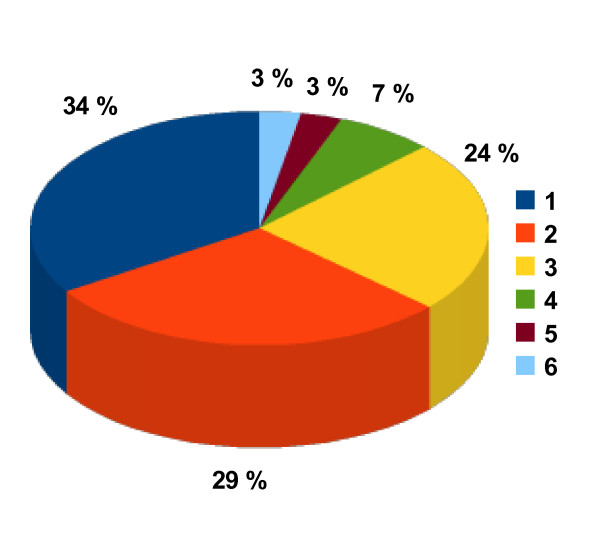
**Fraction [%] of unique reactions in main EC classes (1-6)**.

In Figure 6 the fraction of all unique reactions belonging to the six main *EC *classes is shown. The largest fractions belong to *EC *classes 1 and 2, followed by class 3. Statistical data about the *EC *numbers occurring in the non-redundant reaction database are given in Table 3. Additionally to all *EC *numbers, complete and incomplete, the latter ones are listed separately. Furthermore it is distinguished between *EC *numbers representing at least one single reaction or more than one. A detailed look on the *EC *numbers with the highest number of reactions is given in Table 4 together with the number of reactions.

**Table 3:** Statistics about *EC *numbers occurring in the integrated non-redundant reaction database

*EC *numbers	Different *EC *numbers	Incomplete *EC *numbers
in total	4,288	365
with > 1 reaction	2,681	185
with > 5 reactions	561	73
with > 10 reactions	184	49

**Table 4:** Complete *EC *numbers with the highest number of reactions

*EC *number	Enzyme	Number of reactions
1.14.14.1	unspecific monooxygenase	116
2.4.1.17	glucuronosyltransferase	80
3.2.1.21	*beta*-glucosidase	74
1.1.1.100	3-oxoacyl-[acyl-carrier-protein] reductase	55
3.5.1.4	amidase	46
3.6.3.44	xenobiotic-transporting ATPase	46
3.1.3.16	phosphoprotein phosphatase	44
1.3.1.10	enoyl-[acyl-carrier-protein] reductase (NADPH, B-specific)	43
3.2.1.1	*alpha*-amylase	43
1.1.1.50	3*alpha*-hydroxysteroid dehydrogenase (B-specific)	42
2.3.1.41	*beta*-ketoacyl-acyl-carrier-protein synthase I	41
3.6.1.9	nucleotide diphosphatase	39
1.1.1.1	alcohol dehydrogenase	37
2.3.1.86	fatty-acyl-CoA synthase	37
1.14.13.72	methylsterol monooxygenase	36
1.2.1.3	aldehyde dehydrogenase (NAD^+^)	34
1.2.1.5	aldehyde dehydrogenase [NAD(P)^+^]	33
3.2.1.24	*alpha*-mannosidase	33
3.2.1.51	*alpha-L*-fucosidase	33
1.14.13.8	flavin-containing monooxygenase	32
1.4.3.3	*D*-amino-acid oxidase	32

*Recommended names *of enzymes: source *BRENDA *database.

The only database with a similar goal is *BioSilico *[6]. One important difference consists of the fact that the assignment of identical reactions in our database is done by an actual comparison of the compounds structure in combination with synonyms whereas in *BioSilico*, the matching is only a simple assignment of reactions having the same *EC *number without redundancy check.

The number of reactions in the database described in this paper is far beyond that in *BioSilico*. Selecting three *EC *numbers by chance resulted in *e.g. EC *number *1.14.14.1 *→ 4 reactions in *BioSilico vs*. 116 reactions in our reaction database, *EC *number *2.1.1.103 *→ 1 reaction in *BioSilico vs*. 4 reactions in our database, *3.1.1.47 *→ 1 reaction in *BioSilico vs*. 12 reactions in our database. The fact that in these examples not even all available *KEGG *reactions were found in *BioSilico *indicates that this database is not updated.

Additionally, our reaction database contains the information whether a reaction is stoichiometric incomplete or not. This test is performed before the removal of H^+ ^and H_2_O. Non-balanced reactions are labeled in a separate table column. 2,803 out of 18,172 reactions are at present in this category. The labeling allows modelers to select only balanced reactions for the reconstruction of organism-specific models and networks.

The tool of Radrich *et al. *[10] also includes a stoichiometric evaluation. Their method includes a name comparison where they compare the similarity of compound names. Further they use *SMILES *strings for a structural comparison. The tool was executed only for *Arabidopsis thaliana*, so no general comparison could be done. For this purpose the authors combined data of the databases *KEGG *and *AraCyc *[11].

## Conclusions

In this work we present an integrated and non-redundant reaction database implementing a combined approach of structure and name based comparison.

The tool, integrated into the *BRENDA *[1] query engine but not confined to *BRENDA *data is offering a novel valuable tool that can be used *e.g. *for the construction of biological models. The resulting models will be much more complete than if only one of the databases is used as the three biological databases *BRENDA*, *KEGG *[2], and *MetaCyc *[3] complement each other. Regular 6-monthly updates of this database will make guarantee actuality.

## Availability and requirements

The integrated and non-redundant reaction database is accessible *via BKM-react *[20] and the website of the *BRENDA *[1] database: *BRENDA *website [21] → *Reaction & Specificity *→ *Biochemicals Reactions Aligned *(Figure 1). The complete dataset is additionally provided as a CSV-formatted download at the same site. Available is a reaction table, a table with all compounds occurring in the reactions, and an assignment table with the linkage between reactions and compounds.

## List of abbreviations used

*BRENDA*: *BR*aunschweig *EN*zyme *DA*tabase; *EC*: *E*nzyme *C*ommission; *InChI*: IUPAC *In*ternational *Ch*emical *I*dentifier; *IUBMB*: *I*nternational *U*nion of *B*iochemistry and *M*olecular *B*iology; *IUPAC*: *I*nternational *U*nion of *P*ure and *A*pplied *C*hemistry; *KEGG*: *K*yoto *E*ncyclopedia of *G*enes and *G*enomes; *SMILES*: *S*implified *M*olecular *I*nput *L*ine *E*ntry *S*ystem.

## Authors' contributions

ML and MS executed the data acquisition and implemented the reaction comparison. ML and MS were involved in the construction of the integrated reaction database and the scientific evaluation. DS had the idea to develop the reaction database and supervised the development. ML, MS, and DS wrote the manuscript. All authors read and approved the final manuscript.
